# Supplementary material for: Comparative systems analysis of the secretome of the opportunistic pathogen Aspergillus fumigatus and other Aspergillus species
Source: Sci Rep. 2018 Apr 26;8:6617. doi: 10.1038/s41598-018-25016-4 (PMC5919931; doi:10.1038/s41598-018-25016-4)
Supplement: Supplementary file 1 — Supplementary Text [file 41598_2018_25016_MOESM1_ESM.pdf]

Supplementary Material

for

Comparative systems analysis of the secretome of the opportunistic pathogen *Aspergillus fumigatus* and other *Aspergillus* species

R.P. Vivek-Ananth[1], Karthikeyan Mohanraj[1], Muralidharan Vandanashree[1], Anupam Jhingran[2], James P. Craig[1] and Areejit Samal[1,*]

[1]The Institute of Mathematical Sciences, Homi Bhabha National Institute, Chennai 600113, India.

[2]Stony Brook University, Stony Brook, New York 11794-3369, USA

[*]Corresponding author: asamal@imsc.res.in

Supplementary Text

Comparison of our secretome prediction pipeline with FSD, FunSecKB2 and SECRETOOL

In this section, we compare the secretome predictions from our pipeline with those from previously published pipelines, FSD[1], FunSecKB2[2] and SECRETOOL[3].

FSD[1] relies on nine bioinformatic tools for fungal secretome prediction and its classification into four classes (Supplementary Table S6). While comparing our secretome predictions in *Aspergillus* species with FSD, we have considered the SP class in FSD which corresponds to secreted proteins predicted to have a signal peptide but do not have a transmembrane (TM) domain and an endoplasmic reticulum (ER) retention signal. Note that FSD provides secretome predictions for five *Aspergillus* species analyzed here (Supplementary Table S6). For the *A. fumigatus* strain analyzed here, our pipeline predicts 662 secreted extracellular proteins while FSD predicts 781 secreted proteins in SP class, with 484 secreted proteins in common (Supplementary Table S6). Interestingly, in *A. fumigatus*, one of the secreted proteins in SP class of FSD has intracellular localization based on UniProt annotation with experimental evidence, and therefore, the protein was filtered out in the initial phase of our pipeline (Figure 1 in Main Text). Moreover, in *A. fumigatus*, 99 secreted proteins with experimental evidence from our pipeline were not contained in the set of secreted proteins in SP class of FSD. Furthermore, similar conclusions were obtained while comparing our secretome predictions for *A. niger, A. terreus, A. nidulans* and *A. oryzae* with those in FSD (Supplementary Table S6).

FunSecKB2[2] employs eight bioinformatic tools for fungal secretome prediction and its classification (Supplementary Table S6). Note that FunSecKB2 contains secretome predictions for six *Aspergillus* species analyzed here (Supplementary Table S6). While comparing our secretome predictions in *Aspergillus* species with FunSecKB2, we have considered the set of 'curated' and 'highly likely' secreted proteins in FunSecKB2. For the *A. fumigatus* strain analyzed here, our pipeline predicts 662 secreted proteins while FunSecKB2 predicts 576 secreted proteins, with 496 secreted proteins in common (Supplementary Table S6). Furthermore, in *A. fumigatus*, one of the secreted proteins predicted by FunSecKB2 has intracellular localization based on UniProt annotation with experimental evidence, and therefore, the protein was filtered out in the initial phase of our pipeline (Figure 1 in Main Text). Moreover, similar conclusions were obtained while comparing our secretome predictions for *A. flavus*, *A. niger*, *A. terreus*, *A. nidulans* and *A. oryzae* with those in FunSecKB2 (Supplementary Table S6).

While comparing our secretome predictions in *Aspergillus* species with those from SECRETOOL, we have considered proteins predicted to be secreted by SECRETOOL pipeline using default options on their webserver. For the *A. fumigatus* strain analyzed here, our pipeline predicts 662 secreted proteins while SECRETOOL predicts 365 secreted proteins, with 283 secreted proteins in common (Supplementary Table S6). Furthermore, in *A. fumigatus,* 162 secreted proteins with experimental evidence from our pipeline were not contained in the predicted secretome by SECRETOOL. Moreover, similar conclusions were obtained while comparing our secretome predictions for other *Aspergillus* species analyzed here with those from SECRETOOL (Supplementary Table S6). Thus, SECRETOOL in comparison to our pipeline predicts significantly fewer number of secreted proteins in a fungal genome (Supplementary Table S6).

References

1. Choi, J. *et al.* Fungal secretome database: integrated platform for annotation of fungal secretomes. *BMC Genomics* **11**, 105, doi:10.1186/1471-2164-11-105 (2010).

2. Meinken, J. *et al.* FunSecKB2: a fungal protein subcellular location knowledgebase. *Computational Molecular Biology* **4** (2014).

3. Cortázar, A. R., Aransay, A. M., Alfaro, M., Oguiza, J. A. & Lavín, J. L. SECRETOOL: integrated secretome analysis tool for fungi. *Amino Acids* **46**, 471-473 (2014).
